# Continuous Manufacturing of Cocrystals Using 3D-Printed Microfluidic Chips Coupled with Spray Coating

**DOI:** 10.3390/ph16081064

**Published:** 2023-07-27

**Authors:** Aytug Kara, Dinesh Kumar, Anne Marie Healy, Aikaterini Lalatsa, Dolores R. Serrano

**Affiliations:** 1 Departament of Pharmaceutics and Food Science, School of Pharmacy, Complutense University of Madrid, 28040 Madrid, Spain; akara@ucm.es; 2 Department of Pharmaceutical Engineering & Technology, Indian Institute of Technology, Banaras Hindu University, Varanasi 221001, India; dinesh.phe@itbhu.ac.in; 3 School of Pharmacy and Pharmaceutical Sciences, Trinity College Dublin, D02 PN40 Dublin, Ireland; healyam@tcd.ie; 4 Institute of Pharmacy and Biomedical Sciences, University of Strathclyde, Glasgow G4 0RE, UK; aikaterini.lalatsa@strath.ac.uk; 5 CRUK Formulation Unit, School of Pharmacy and Biomedical Sciences, University of Strathclyde, Glasgow G4 0RE, UK; 6 Institute of Industrial Pharmacy, School of Pharmacy, Complutense University of Madrid, 28040 Madrid, Spain

**Keywords:** cocrystal, SLA, microfluidics, chips, 3D printing, spray coating, fluidized bed, sulfadimidine, 4-aminosalicylic acid, continuous manufacturing, crystal habit

## Abstract

Using cocrystals has emerged as a promising strategy to improve the physicochemical properties of active pharmaceutical ingredients (APIs) by forming a new crystalline phase from two or more components. Particle size and morphology control are key quality attributes for cocrystal medicinal products. The needle-shaped morphology is often considered high-risk and complex in the manufacture of solid dosage forms. Cocrystal particle engineering requires advanced methodologies to ensure high-purity cocrystals with improved solubility and bioavailability and with optimal crystal habit for industrial manufacturing. In this study, 3D-printed microfluidic chips were used to control the cocrystal habit and polymorphism of the sulfadimidine (SDM): 4-aminosalicylic acid (4ASA) cocrystal. The addition of PVP in the aqueous phase during mixing resulted in a high-purity cocrystal (with no traces of the individual components), while it also inhibited the growth of needle-shaped crystals. When mixtures were prepared at the macroscale, PVP was not able to control the crystal habit and impurities of individual mixture components remained, indicating that the microfluidic device allowed for a more homogenous and rapid mixing process controlled by the flow rate and the high surface-to-volume ratios of the microchannels. Continuous manufacturing of SDM:4ASA cocrystals coated on beads was successfully implemented when the microfluidic chip was connected in line to a fluidized bed, allowing cocrystal formulation generation by mixing, coating, and drying in a single step.

## 1. Introduction

Drug delivery systems, advanced manufacturing techniques, and the discovery of new active pharmaceutical ingredients (APIs) have contributed to significant advancements in the pharmaceutical industry over the past few decades. Cocrystals have shown great promise in enhancing the physicochemical properties of APIs, such as solubility, stability, and bioavailability. Cocrystals are crystalline materials composed of two or more molecular components, typically an active pharmaceutical ingredient (API) and a co-former, held together by non-covalent interactions such as hydrogen bonding, van der Waals forces, and Π-stacking [1]. The formation of cocrystals can significantly alter an API’s physicochemical properties, such as solubility, dissolution rate, stability, and bioavailability, without affecting its pharmacological activity [2]. Selecting suitable co-formers is a crucial aspect of cocrystal design, significantly affecting its overall performance. Co-formers are typically chosen based on their ability to form non-covalent solid interactions with the API, their solubility in common solvents, their chemical stability and compatibility with the API, and their regulatory approval [3]. In recent years, cocrystals have garnered much attention due to their potential to address numerous challenges associated with developing new drug formulations, such as poor API solubility and bioavailability. Cocrystals have been studied widely for oral drug delivery because they improve poorly soluble drugs’ solubility and dissolution rates [4]. However, the potential of cocrystals for non-oral administration has remained relatively unexplored.

**Figure 1:**
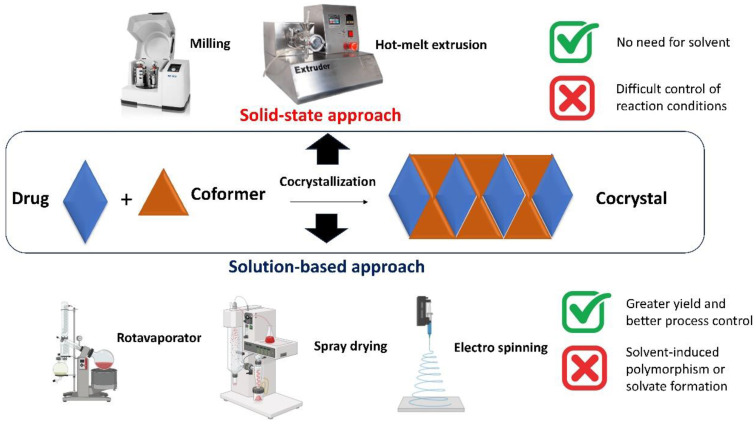
Comparison of cocrystal formation using solid-state and solution-based methods.

The production of cocrystals can be broadly divided into solid-state and solution-based techniques (Figure 1). Solid-state methods involve the mechanical grinding or milling of the API and co-former in their solid forms, either manually or with specialized types of machinery, such as ball and jet mills and hot-melt extrusion [5,6,7,8,9]. Solid-state methods have the advantage of not requiring solvents. However, their difficulty in controlling the reaction conditions, such as temperature and pressure, often results in low yields and poor reproducibility. In contrast, solution-based methods involve the dissolution of the API and co-former in an appropriate solvent, followed by the precipitation or crystallization of the cocrystal via solvent evaporation, cooling, or the addition of an antisolvent [10,11]. Solution-based methods typically provide greater yields and control over the reaction conditions than solid-state methods. However, they are frequently accompanied by longer processing times, increased energy consumption, and the possibility of solvent-induced polymorphism or solvate formation [12]. In recent years, novel alternative methods to fabricate cocrystals have emerged, such as hot-melt extrusion, spray drying, and electrospinning, which offer the potential for continuous manufacturing and process intensification, enhancing control of the reaction conditions [7,11,13]. These techniques are still in their infancy and require additional optimization and validation before the pharmaceutical industry can adopt them.

Microfluidic technology has emerged as a promising alternative to conventional cocrystal production techniques due to its capacity to provide precise control over reaction conditions, rapid mixing, and efficient heat transfer, which can significantly improve the quality and yield of cocrystals [14]. Lab-on-a-chip systems, or microfluidic devices, consist of microscale channels and chambers through which small volumes of fluids can be manipulated and processed with high precision and reproducibility [15]. Mixing the API and co-former solutions in a microchannel, followed by the controlled precipitation or crystallization of the cocrystal in a downstream section of the device, is a technique that has been previously applied to cocrystal screening of pharmaceutical parent compounds [14]. The potential of microfluidic technology has already been demonstrated for the continuous production of cocrystals, including carbamazepine–nicotinamide, theophylline–citric acid, and indomethacin–saccharin cocrystals [16]. Significant improvements have been found in the yield, purity, and physicochemical properties of cocrystals prepared using microfluidics compared to conventional manufacturing methods, highlighting the potential of microfluidic technology for the pharmaceutical industry’s large-scale production of cocrystals.

Microfluidic chips have several advantages over conventional techniques such as solvent evaporation, grinding, and slurry crystallization in the fabrication of cocrystals [17]. Microfluidic devices enable precise control over the flow rates, mixing ratios, and temperature of the API and co-former solutions, which substantially affects the formation and quality of cocrystals. The small dimensions of the microchannels allow rapid mixing and efficient heat transfer, which helps in the formation of high-quality cocrystals with narrow size distributions and reduced batch-to-batch variation [18]. Additionally, microfluidic technology enables continuous cocrystal production, which is more efficient than batch-based techniques, typically requiring smaller volumes of solvents than conventional methods, which can reduce the environmental impact and cost of the manufacturing process. Furthermore, microfluidic technology can be easily customized and integrated with other analytical or processing modules, such as in-line monitoring systems, to facilitate real-time process control and optimization.

Despite these advantages, microfluidic chip production faces limitations and challenges that must be overcome. For instance, microfluidic device fabrication can be difficult and expensive, especially for mass production. In addition, the small dimensions of the microchannels can lead to clogging and fouling issues, affecting the performance and dependability of the device. Furthermore, scaling up microfluidic processes for industrial applications remains challenging due to the difficulty of maintaining the same degree of control and efficiency at larger scales [17]. However, many of these barriers can be overcome by 3D printing, which is more cost-effective than other conventional techniques to fabricate microfluidic chips with great channel resolution and a variety of materials [19].

Three-dimensional printing technologies are divided into seven categories. However, only two techniques, fuse deposition modeling (FDM) and stereolithography (SLA/DLP), have shown promising results when printing microfluidic chips, taking into account the high resolution needed for printing the micrometric channels [19]. The main difference observed between 3D-printed microfluidic chips using FDM techniques and those using SLA techniques is the roughness of the internal channels. SLA 3D-printed chips have shown less rough internal surface channels compared to FDM chips, which can alter the mixing flow properties [20].

The hypothesis underpinning this work is that 3D-printed microfluidic chips using SLA techniques can be applied in the continuous manufacturing of cocrystals, allowing process intensification and better control of particle characteristics than conventional methods. This would favor the use of cocrystals for administration routes other than just oral. For the first time, continuous manufacturing of cocrystals using 3D-printed microfluidic chips coupled with a fluidized bed has been developed. Sulfadimidine (SDM)-4-aminosalicylic acid (4ASA) was used as a model cocrystal, which can be used for the combined treatment of infection and inflammation [21]. Previously, we have demonstrated that this cocrystal can be coated on beads using a fluidized bed [22]. To ensure an optimal coating process, the cocrystal components should be co-dissolved with a binder agent such as polyvinyl pyrrolidone (PVP). However, it is well-known that PVP, due to its amorphous nature, can trigger amorphization, preventing crystallization [23]. Controlling process parameters before spray coating is crucial to ensure optimal cocrystallization. In this work, continuous manufacturing of SDM:4ASA cocrystal combined with PVP as a binder will be investigated using a novel 3D-printed microfluidic device coupled with a fluidized bed.

## 2. Results

### 2.1. Engineering of 3D-Printed Microfluidic Chips

**Figure 2:**
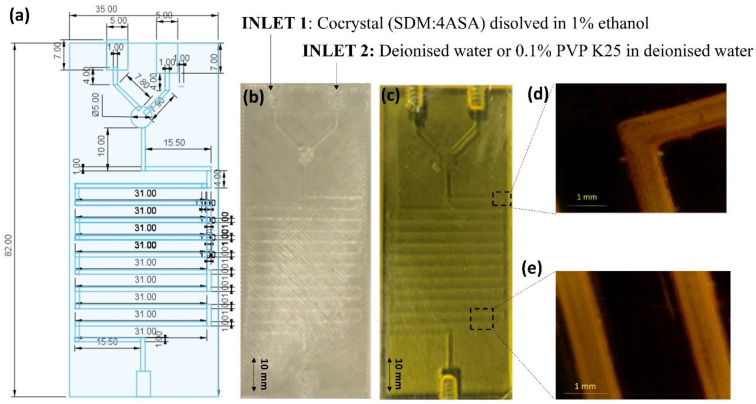
Engineering and manufacturing of 3D-printed microfluidic chips (adapted from [20]). Key: (**a**) Geometrical design of the 3D-printed microfluidic chip; units expressed in millimeters; (**b**) Digital photograph of the 3D-printed microfluidic chip by SLA; (**c**) Optical microscope image of SLA microfluidic chip channel curvature; (**d**,**e**) Optical microscope image of the longitudinal channel and corner of the SLA microfluidic chip.

**Figure 3:**
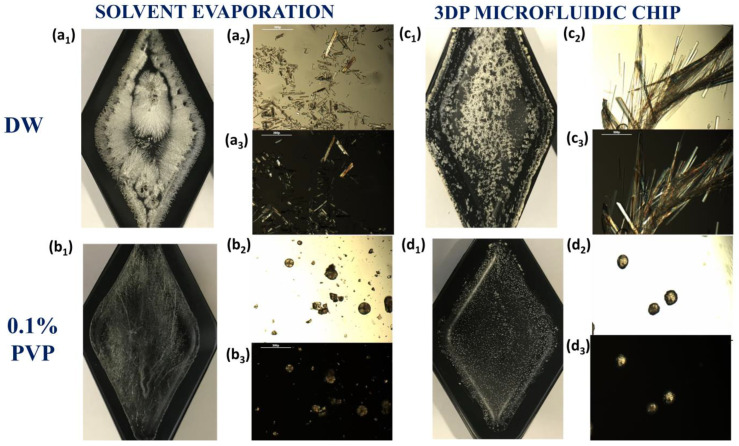
Morphological evaluation of cocrystals manufactured by macroscale solvent evaporation (**a**,**b**) or mixed in the 3D-printed (3DP) microfluidic chips (**c**,**d**). Key: DW, deionized water. Cocrystals prepared using solvent evaporation with deionized water (DW) (**a**) or 0.1% PVP K25 in deionized water (**b**) as the antisolvent; cocrystals prepared with the microfluidic chip with deionized water (**c**) or 0.1% PVP K25 in deionized water (**d**) as the antisolvent; images obtained from an iPhone camera (1), microscopy image at 20× *g* magnification (1); (3) polarized optical image at 20× *g* magnification.

Microfluidic chips were printed using an SLA printer exhibiting the same geometry as the CAD design (Figure 2). Channels were fully opened after the washing and post-curing process. The average surface roughness of the channels was 0.156 ± 0.106 µm [20]. The channels of the microfluidic chip were designed with 1 mm in diameter, which exactly corresponds to the visualized channels in the optical microscope (Figure 3c,d). Both the linear part of the channels and the corners showed an excellent resolution compared to the CAD design.

### 2.2. Cocrystal Morphological Characterization

The crystal habit morphology was significantly different between the cocrystals manufactured using the microfluidic chip and those manufactured by solvent evaporation (Figure 3). When cocrystals were formed using deionized water (DW) as the antisolvent, long needle-shape birefringent crystals were observed using the optical microscope (Figure 3a,c). However, the presence of 0.1% PVP K25 in the antisolvent aqueous solution inhibited the crystal growth, resulting in spherical birefringent cocrystals. The process was more homogenous when the mixture took place inside the microfluidic chip (Figure 3d), as conventional solvent evaporation resulted in a mixture of spherical and needle-shape crystals (Figure 3b).

**Figure 4:**
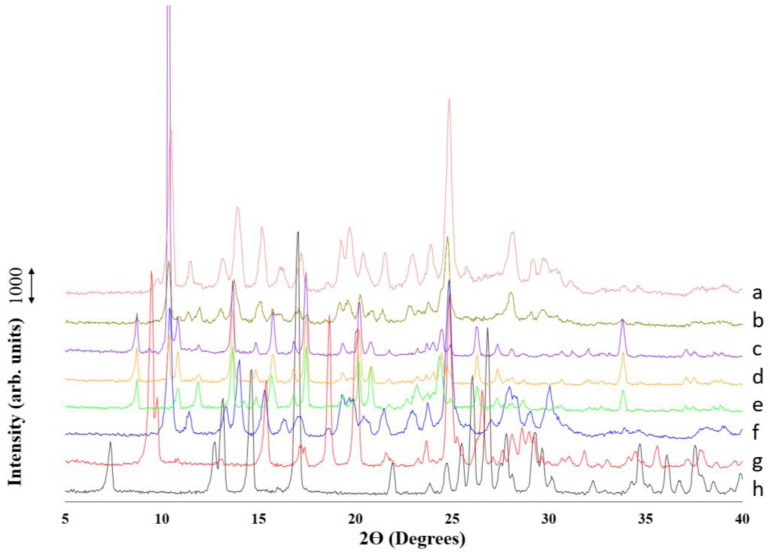
PXRD pattern of cocrystals fabricated using macroscale solvent evaporation or mixing in the 3D-printed microfluidic chip and single components. a: Solvent evaporation with 0.1% PVP K25; b: microfluidics with 0.1% PVP K25; c: solvent evaporation with deionized water; d: microfluidics with deionized water; e: cocrystal polymorph II produced under rota evaporation as comparison; f: cocrystal polymorph I produced using liquid-assisted ball milling; g: SDM raw unprocessed material; h: 4ASA raw unprocessed material.

Notable differences were also observed in the PXRD pattern of the dried cocrystals (Figure 4). Two different polymorphs have been described for 4ASA: SDM cocrystals [21]. The manufacturing method significantly affects the formation of one or another polymorph as well as the crystal habit [24]. When deionized water was used as an antisolvent, the cocrystal formed exhibited Bragg peaks attributed to both polymorph I and polymorph II. This is visible in the peaks at 10.5 2 θ degrees, attributed to polymorph I, and at 10.8 2 θ degrees, attributed to polymorph II (Figure 4c,d). However, traces of SDM at 9.4 2 θ degrees were also observed when the cocrystal was manufactured using the solvent evaporation method but not the microfluidic method. When 0.1% PVP K25 aqueous solution was used as an antisolvent, not only was the cocrystal habit altered, but a polymorphic transition towards polymorph I also occurred. Similarly, traces of SDM at 9.85 2 θ degrees were observed only in the cocrystal formed using solvent evaporation, not with the 3D-printed microfluidic chip.

**Figure 5:**
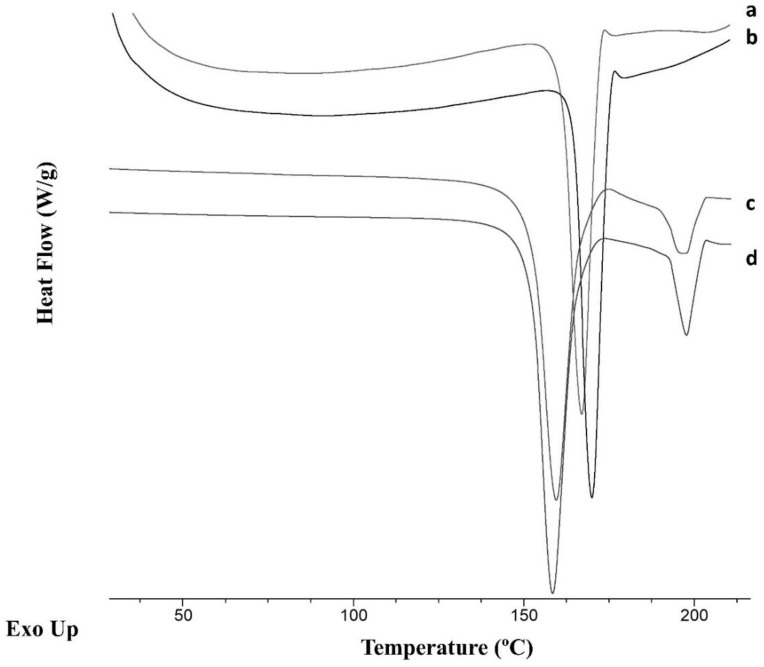
DSC thermograms of cocrystals fabricated using solvent evaporation or microfluidic mixing using deionized water. Key: microfluidic mixing (a), macroscale solvent evaporation (b), solvent evaporation using rota evaporation resulting in polymorph II (c), and liquid-assisted ball milling resulting in polymorph I (d).

The thermograms of cocrystals produced using the 3D-printed microfluidic chip as well as those manufactured by conventional formulation were compared with the cocrystal polymorph II produced under rota evaporation and cocrystal polymorph I produced using liquid-assisted ball milling [24]. The melting point of the cocrystal was found between the previously reported melting points of the single components (SDM, 197.1 °C and 4ASA, 145.6 °C) [24]. The melting points of the cocrystals were 152.6 °C and 152.3 °C for polymorph I and II, respectively [22]. When polymorph I and II cocrystals were manufactured using liquid-assisted ball milling and rota evaporation, a secondary peak located at temperatures similar to the SDM single component was also observed, which can be related to traces of SDM in the solid powder mixture. Surprisingly, a significant shift in the onset melting point occurred when the cocrystals were manufactured in the presence of deionized water, both using 3D-printed microfluidic chips (161.27 ± 0.4 °C) and using conventional solvent evaporation (164.7 ± 0.5 °C) (Figure 5). No secondary peak at 197 °C was observed in either case, indicating a lower degree of traces of SDM. The enthalpy of fusion was similar for cocrystals produced by macroscale solvent evaporation using deionized water (219.8 ± 0.9 J/g) as antisolvent and those produced microfluidically (212.3 ± 2.4 J/g). However, the enthalpy was lower compared to cocrystals produced by liquid-assisted ball milling or rota evaporation (260.8 ± 1.2 J/g and 249.7 ± 0.7 J/g for polymorph I and II, respectively).

**Figure 6:**
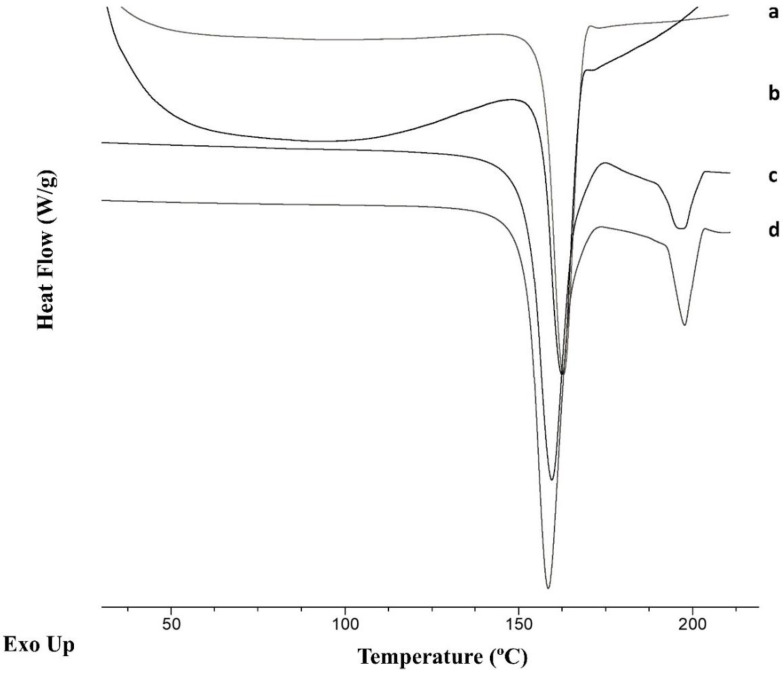
DSC thermograms of cocrystals fabricated using solvent evaporation or mixing in the 3D-printed microfluidic chip using 0.1% PVP K25. Key: microfluidic mixing (a), solvent evaporation (b), solvent evaporation using rota evaporation resulting in polymorph II (c), and liquid-assisted ball milling resulting in polymorph I (d).

A decrease in the melting point as well as in the enthalpy of fusion was observed for the cocrystals manufactured using 0.1% PVP K25 (Figure 6). The onset melting point for the cocrystal manufactured using the 3D-printed microfluidic chips and 0.1% PVP K25 was 157.2 ± 0.6 °C, with an enthalpy of fusion of 201.5 ± 2.4 J/g, while the onset melting point for the cocrystal manufactured by solvent evaporation was 156.4 ± 0.9 °C, with a 170.6 ± 1.5 J/g enthalpy and a pronounced dehydration peak. A secondary peak at 197 °C attributed to SDM was not observed in either case. The decrease in the melting point could also be attributed to a partial drug amorphization.

### 2.3. Continuous Manufacturing of Cocrystals Using a Solution-Based Microfluidic Approach Coupled with Spray Coating

**Figure 7:**
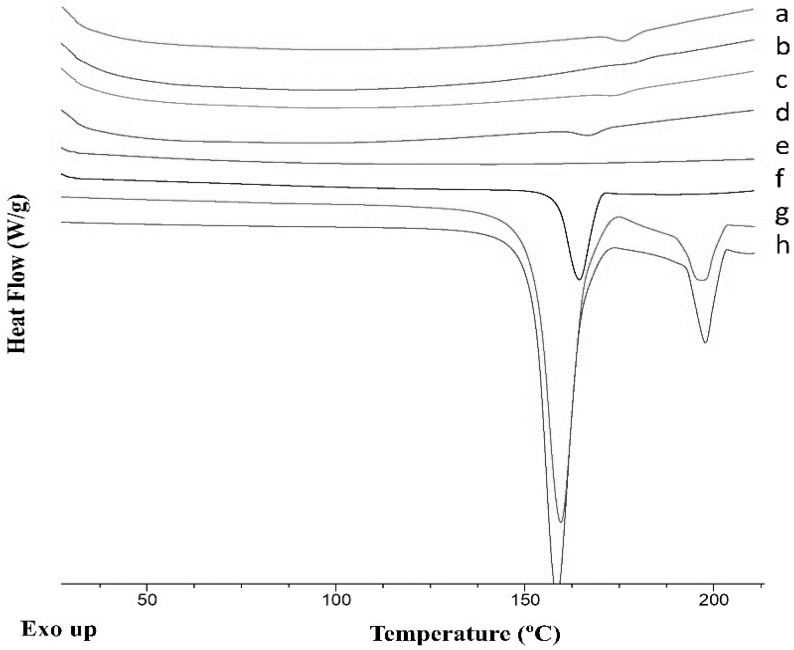
DSC thermograms of cocrystals after spray coating, compared to those prepared by rota evaporation or liquid-assisted ball-milling. Key: Cocrystals microfluidically prepared using deionized water (a), cocrystals mixed at the macroscale (b), cocrystals microfluidically prepared using 0.1% PVP 25 (c); cocrystals mixed at the macroscale prepared using 0.1% PVP 25 in deionized water mixed with ethanol (d), blank MCC beads (e), MCC beads coated with an ethanolic solution containing 5% PVP K90 and 95% cocrystal components (f); SDM:4ASA cocrystal polymorph II obtained by conventional solvent evaporation from ethanol (g); SDM:4ASA cocrystal polymorph I obtained by liquid-assisted ball milling (h).

**Figure 8:**
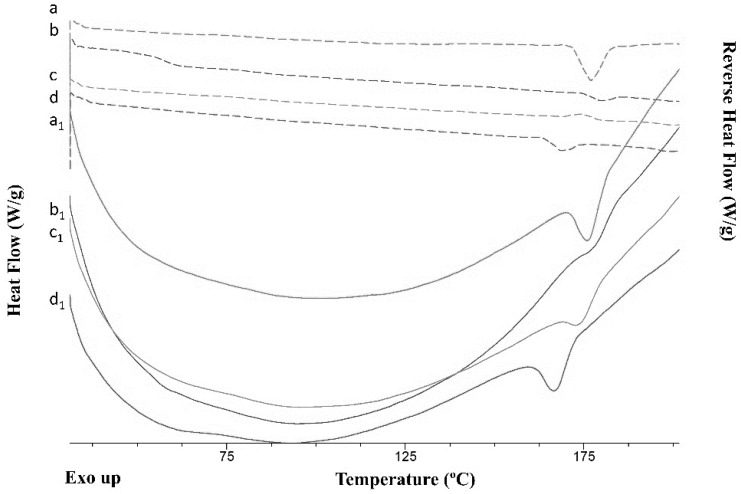
DSC thermograms of cocrystals after spray coating. Key: Thermograms a–d indicate heat flow and a_1_–d_1_ indicate reversing heat flow thermograms; cocrystal components mixed microfluidically using deionized water (a, a_1_), macroscale mixing in ethanol: water (1:1, *v*:*v*) (b, b_1_) microfluidically using 0.1% PVP 25 (c, c_1_), and macroscale mixing using 0.1% PVP 25 (d, d_1_).

The yield of the process obtained using the 3D-printed microfluidic chips (method 2) was similar to that obtained by conventional mixing (method 1) [22]. The melting point and the onset of the melting point of the cocrystals were observed in all the thermograms (Figure 7) irrespective of whether deionized water or 0.1% *w*/*v* PVK K25 was used. The enthalpy of fusion for the cocrystals obtained by continuous manufacturing (4 J/g) (methods 2 and 3) was lower than for those obtained by the conventional spray coating method (49 J/g) (method 1) with a lower ratio of cocrystal components: MCC beads were used (1:4 versus 1:1.2). The melting point and the enthalpy of fusion for cocrystals manufactured microfluidically using deionized water as antisolvent were 170.8 ± 0.9 °C and 4.3 ± 0.2 J/g respectively (method 2), while cocrystals dissolved in ethanol and deionized water sprayed directly onto the beads (method 3) showed a melting at 172.7 ± 0.9 °C and a heat of fusion of 1.1 ± 0.2 J/g. The latter showed a clear glass transition in the reversing heat flow signal at 57.2 ± 1.5 °C. This indicates the presence of traces of free SDM, which is aligned with the PXRD results (Figure 8). When using 0.1% PVP K25 aqueous solution, cocrystals prepared microfluidically showed an onset melting temperature at 168.4 ± 2.4 °C and a fusion enthalpy of 2.1 ± 0.2 J/g (method 2). However, the same mixture component directly sprayed onto the beads exhibited melting at 160.9 ± 0.9 °C and an enthalpy of 4.0 ± 0.5 J/g (method 3).

## 3. Materials and Methods

### 3.1. Materials

SDM and 4ASA with a purity ≥ 99%, were purchased from Sigma–Aldrich (Wicklow, Ireland). Ethanol was supplied from Corcoran Chemicals (Dublin, Ireland). Cellets^®^ (500 µm pellets made of microcrystalline cellulose) were a gift from Pharmatrans Sanaq AG (Allschwi, Switzerland). Polyvinyl pyrrolidone (PVP K25) was a gift from BASF (Ludwigshafen, Germany). UV polymerisable resin (405 nm) was obtained from Anycubic^®^ (Shenzhen, China). Anycubic Photon Mono X (LCD-based SLA printer, 405 nm light source, 0.05 mm 3840 × 2400 XY resolution, 0.01 mm Z resolution, 192 × 120 × 245 mm build volume) and Anycubic Mega Zero (FDM printer, 0.1 mm layer resolution, 0.125 mm XY resolution, 0.4 mm nozzle diameter) were purchased from Anycubic^®^ (Shenzhen, China).

### 3.2. Methods

#### 3.2.1. Design and 3D Printing of Microfluidic Chips

##### Geometrical Design

The microfluidic chips were designed as previously described [20] using Tinkercad^®^ (Autodesk^®^, San Francisco, CA, USA), with a total length of 8.2 cm, a width of 3.5 cm, and a height of 0.7 cm. Each microfluid chip was designed with two inlets consisting of two inlet channels (2 cm in length and 1 mm diameter) leading to a circular chamber, followed by a radiator shape channel (44 cm in length, 1 cm of internal diameter). The 3D design was exported into a standard tessellation language (.stl) digital file, which was imported into Anycubic Photon Slicer Software (Anycubic^®^, Shenzhen, China). The .stl file was sliced into a .pwmx file readable by the SLA printer.

##### Stereolithography (SLA)

Chips were printed using an Anycubic^®^ Photon Mono X SLA printer with a photocurable resin (Anycubic^®^ UV sensitive resin transparent yellow) polymerized at 405 nm. The thickness of each layer was 50 µm, resulting in 140-layer chips. The first eight layers were cured for 60 s to ensure an optimal attachment to the metallic platform of the printer, while the rest of the layers were cured just for 3 s to avoid over-curing and channel blocking. After printing, channels were washed out with 96% ethanol, and the chip was cured for 120 min with Anycubic post-curing equipment (Anycubic Wash & Cure plus, Anycubic, Shenzhen, China).

##### Imaging

After printing, the geometry of the chips was visualized with an iPhone 10 12-megapixel camera (f/1.8, 1.22-micron) (Apple, Cupertino, CA, USA) and an optical digital microscope (1000×) using cooling tech software (Turejo Comp. Guangzhou, China). The geometry of the chips was visualized with a ZEISS Primo Star microscope (Carl Zeiss Microscopy, White plains, NY, USA) eyepiece magnification of 10× *g* and objective magnification of 4× *g*.

#### 3.2.2. Preparation of Cocrystal Formulations

All cocrystals (SDM:4ASA) were prepared using a 1:1 molar ratio as previously described [24,25].

##### Macroscale Solvent Evaporation

4ASA (153.4 mg) and SDM (278.3 mg) were dissolved in 50 mL of ethanol (1% solution). The ethanolic solution was mixed in a vial with either deionized water (DW) or 0.1% PVP K25 aqueous solution. One milliliter of these mixtures was transferred into black plastic weighing boats, and the solvent was allowed to evaporate overnight at 20 °C.

##### Microfluidic Mixing

An ethanolic solution containing 4ASA (153.4 mg) and SDM (278.3 mg) in 50 mL of ethanol (1% solution) was prepared and pumped using a peristaltic pump at a speed of 0.625 mL min^−1^ via one of the inlets of the microfluidic chip. The second inlet was fed either with deionized water or 0.1% PVP K25 aqueous solution pumped at the same speed (0.625 mL min^−1^). The eluate was collected through the outlet port and, similar to the conventional method, one milliliter of the liquid mixture was transferred into a black plastic weighing boat and was left to dry overnight at 20 °C.

##### Imaging

The morphology of the dried powder collected after solvent evaporation and mixing in the microfluidic chip was performed using a 12MP iPhone 10 camera. An Olympus BX35 (Tokyo, Japan) upright polarizing microscope and Lynksys 32 software were also used to visualize in detail the morphology of the cocrystals formed by conventional solvent evaporation or after mixing within the 3D-printed microfluidic chips [26].

##### Powder X-ray Diffraction

Cocrystals prepared by macroscale or microfluidic mixing and evaporation were compared to large plate cocrystals produced by solvent evaporation using a rota evaporator (Buchi, Flawil, Switzerland) at 250 mbar pressure and 55 °C as previously described [24] and prismaticcocrystals produced by liquid-assisted co-milling carried out in a planetary ball mill (Retsch PM100, Haan, Germany) as previously described [21].

Powder X-ray analysis was performed using a Miniflex II Rigaku diffractometer with Ni-filtered Cu Kα radiation (1.54 Å) (Tokyo, Japan). The tube voltage and tube current used were 30 kV and 25 mA, respectively. The PXRD patterns were recorded (*n* = 3) from 5° to 40° on the 2 theta scale at a step scan rate of 0.05° per second [24].

#### 3.2.3. Continuous Manufacturing of Cocrystals Using a Solution-Based Microfluidic Approach Coupled with Spray Coating

##### Method 1

In a previous study, a 5 g batch of the same formulation was prepared using 500 µm microcrystalline (MCC) starter cores (2.75 g) coated with an ethanolic solution (225 mL) that contained 5% PVP K90 (0.1125 g) and 95% cocrystal components (1.375 g of SDM and 0.758 g of 4ASA) [22]. The ethanolic solution was sprayed using an inlet temperature of 60 °C via a 0.5 mm nozzle diameter at a 2.5 g min^−1^ spray rate, 25 m^3^ h^−1^ nitrogen flow rate, and a 0.75 bar atomization pressure.

##### Method 2

**Figure 9:**
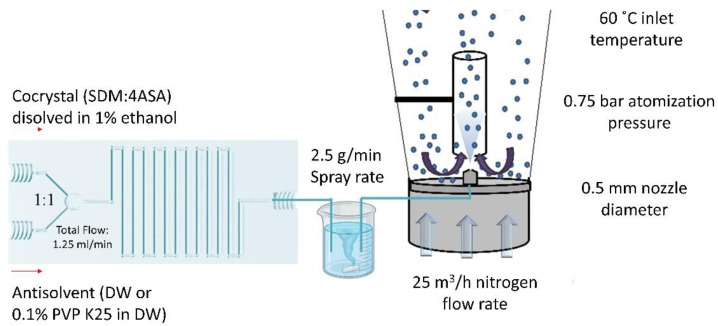
Schematic representation of the continuous manufacturing of cocrystal-coated beads. Key: DW, deionized water.

In this work, a continuous manufacturing method for cocrystal-coated beads involving a 3D-printed microfluidic chip coupled with a fluidized bed (Figure 9) was utilized.

A 5 g batch was manufactured, consisting of 4 g of MCC beads and 1 g of eluate after microfluidic mixing. As the total flow rate used in the microfluidic chip was 1.25 mL min^−1^, which is half the minimum spray rate for the fluidized bed (2.5 g min^−1^), an intermediate container was placed between the chip and the fluidized bed, acting as a low-pressure reservoir under stirring (250 rpm). Two different experiments were performed, adding either (1) deionized water through the second inlet of the chip or (2) an aqueous solution with 0.1% *w*/*v* PKP K25. In the first experiment, deionized water (100 mL) was pumped through one of the inlet ports of the microfluidic chip, while 1% cocrystal mixture (355.3 mg 4ASA and 644.6 mg SDM) dissolved in ethanol (100 mL) was pumped through the other inlet. Each line was mixed in a 1:1 (*v*/*v*) ratio, resulting in a total flow rate of 1.25 mL min^−1^. In the second experiment, the deionized water was replaced by an aqueous solution (100 mL) containing 0.1% *w*/*v* of PVP K25 (100 mg).

##### Method 3

For comparison purposes, the same mixtures of cocrystal dissolved in ethanol and deionized water or 0.1% PVP in water were fed directly into the inlet port of the fluidized bed and sprayed at the same conditions as above described in Figure 2.

##### Differential Scanning Calorimetry

MTDSC scans were performed on a QA-200 TA instrument (TA instruments, Elstree, United Kingdom) calorimeter using nitrogen as the purge gas. Powder materials and intact beads were weighed (3–5 mg) and sealed in closed aluminum pans with one pinhole. A scanning rate of 5 °C/min with an amplitude of modulation of 0.796 °C and modulation frequency of 1/60 Hz were utilized. The temperature range was from 25 °C to 210 °C [27]. Calibration of the instrument was carried out previously using indium as standard. Temperatures of melting events (*n* = 3) refer to onset temperatures [22].

## 4. Discussion

The continuous manufacturing of cocrystals using a solution-based microfluidic approach coupled with spray coating was successful, allowing more precise control of particle formation and polymorphism. The results reveal that the crystal habit of cocrystals manufactured using the microfluidic chip differed significantly from those formed by solvent evaporation.

Particle size and morphology control are crucial for industrial manufacturing. The needle-shaped morphology is often considered high-risk and a source of difficulty in the manufacture of solid dosage forms. Needle-shaped crystals often have a compromised flowability, impacting processes such as hopper discharge, die filling, and other volumetric dosing operations, as well as poorer compactability properties [28,29,30]. Additionally, the needle-shaped morphology also implies tremendous difficulties for particle characterization techniques such as laser diffraction, which assumes spherical particles [31]. Given the inherent undesirability of needle-shaped crystals in pharmaceutical processes, different approaches are adopted to transform them into more amenable crystal habits, typically focused on the addition of additives to alter the growth behavior during crystallization or solvent manipulation [24,32]. However, crystal habit modification can impact cocrystal solubility, yield, and impurity formation [29].

The nucleation rate of cocrystals can be controlled using microfluidic chips. However, the nucleation rate is determined by experimental conditions such as the ratio of co-formers, co-former concentration, and temperature [33]. Significant differences have been found in the SDM:4ASA cocrystal habit between conventional mixing techniques and microfluidic devices. In the current work, the ratio of API: co-former was kept constant (1:1 molar ratio), as previously described by other authors [21,24]. We hypothesize that, in our work, the cocrystal nucleation was not initiated within the chip, as the eluate was transparent. This can be related to the lower concentration of API and co-former in the ethanolic phase as well as the diameter of the channels and the lack of cooling conditions. Further investigations should be performed to modulate these parameters if controlled nucleation inside the microfluidic chip is desired. However, the mixing of both aqueous and ethanolic phases inside the microfluidic channels had a significant impact on cocrystal habit and polymorphism. The addition of PVP in the aqueous phase during mixing resulted in an effective crystal growth inhibition when the mixing occurred in the microfluidic device, which can be attributable to a more homogenous and rapid mixing process due to the precise control over the flow conditions and the high surface-to-volume ratios within the microchannels of the chip. This alteration in crystal habit morphology can be explained by the adsorption of PVP on the crystal surface, inhibiting the growth of specific crystal faces and promoting the formation of spherical cocrystals.

Previously, the crystal-growth-inhibitory effect of PVP on indomethacin crystals in an aqueous medium has been demonstrated [34]. This effect is related to the extent of PVP adsorption on indomethacin crystals, leading to a change in the rate-limiting step from bulk diffusion to surface integration. Also, a great barrier for surface diffusion of indomethacin is provided by a more viscous layer of PVP [34]. We hypothesize that similar crystal growth inhibition takes place in the ethanolic–aqueous mixture of the PVP: SDM:4ASA cocrystal.

The combination of PVP with the mixing of cocrystal components inside microfluidic chips triggered the formation of polymorph I with no traces of individual components (SDM), as occurred during conventional mixing. Microfluidic chips in combination with additives such as PVP have demonstrated a superior capacity for cocrystal engineering in terms of crystal habit manipulation and purity. This is a major advantage that requires further investigation, as it may facilitate the manufacturing of cocrystals for different administration routes, such as oral delivery in powder form or coated pellets, but also for parenteral administration upon powder reconstitution [11,22].

Also, this is the first time that the feasibility of the continuous manufacturing of cocrystals using 3D-printed microfluidic chips coupled with a fluidized bed has been demonstrated. This system could be easily implemented within other drying techniques, such as spray drying. An adjustment of mass transfer should be optimized between the eluate of the chip and the liquid entrance to the fluidized bed to ensure a fully closed continuous manufacturing system. Process intensification is crucial to the pharmaceutical industry minimizing cost and controlling particle engineering and final product characteristics. Further investigation should be performed to implement process-analytical technologies (PAT) during manufacturing, such as including NIR probes in the fluidized bed to quantify the amount of solvent remaining and determine the final point of the drying step during the coating process [19,35,36], to create a robust and highly controlled process.

Another point of concern is 3D printing implementation on a large global scale, taking into account the accessibility of equipment and reagents. Costs associated with SLA printers have been reduced drastically over the last few years. This factor, combined with the fast printing speed of SLA printers, normally requiring less than 3–4 h to print a chip, makes this technology affordable. However, photopolymerizable resins used in the manufacturing of the chips can show lixiviation issues, and hence, unreacted polymers could pass through the solution, especially when stronger solvents are used, resulting in undesirable side effects. A post-curing printing step is critical to minimize this issue, which should be investigated in detail before use in clinical practice.

## 5. Conclusions

SDM:4ASA cocrystal particle engineering has been successfully achieved using 3D-printed microfluidic chips. The addition of PVP in the aqueous phase during mixing has allowed the inhibition of needle-shaped crystals and the generation instead of spherical crystal habits with higher purity compared to conventional mixing. A successful continuous manufacturing method for the fabrication of cocrystal-coated particles has been demonstrated by the combination of microfluidic chips with a fluidized bed, allowing the process intensification of mixing and drying in one step.

## Data Availability

Data will be made available upon request.
